# *De novo* sequencing and analysis of the transcriptome during the browning of fresh-cut *Luffa cylindrica* 'Fusi-3' fruits

**DOI:** 10.1371/journal.pone.0187117

**Published:** 2017-11-16

**Authors:** Haisheng Zhu, Jianting Liu, Qingfang Wen, Mindong Chen, Bin Wang, Qianrong Zhang, Zhuzheng Xue

**Affiliations:** 1 Crops Research Institute, Fujian Academy of Agricultural Sciences, Fuzhou, Fujian, China; 2 Vegetable Research Center, Fujian Academy of Agricultural Sciences, Fuzhou, Fujian, China; 3 Fujian Engineering Research Center for Vegetables, Fuzhou, Fujian, China; Youngstown State University, UNITED STATES

## Abstract

Fresh-cut luffa (*Luffa cylindrica*) fruits commonly undergo browning. However, little is known about the molecular mechanisms regulating this process. We used the RNA-seq technique to analyze the transcriptomic changes occurring during the browning of fresh-cut fruits from luffa cultivar ‘Fusi-3’. Over 90 million high-quality reads were assembled into 58,073 Unigenes, and 60.86% of these were annotated based on sequences in four public databases. We detected 35,282 Unigenes with significant hits to sequences in the NCBInr database, and 24,427 Unigenes encoded proteins with sequences that were similar to those of known proteins in the Swiss-Prot database. Additionally, 20,546 and 13,021 Unigenes were similar to existing sequences in the Eukaryotic Orthologous Groups of proteins and Kyoto Encyclopedia of Genes and Genomes databases, respectively. Furthermore, 27,301 Unigenes were differentially expressed during the browning of fresh-cut luffa fruits (i.e., after 1–6 h). Moreover, 11 genes from five gene families (i.e., *PPO*, *PAL*, *POD*, *CAT*, and *SOD*) identified as potentially associated with enzymatic browning as well as four WRKY transcription factors were observed to be differentially regulated in fresh-cut luffa fruits. With the assistance of rapid amplification of cDNA ends technology, we obtained the full-length sequences of the 15 Unigenes. We also confirmed these Unigenes were expressed by quantitative real-time polymerase chain reaction analysis. This study provides a comprehensive transcriptome sequence resource, and may facilitate further studies aimed at identifying genes affecting luffa fruit browning for the exploitation of the underlying mechanism.

## Introduction

*Luffa cylindrica* (i.e., luffa) of the family Cucurbitaceae is one of the most important vegetables and widely used medicinal plants in China. People are increasingly starting to pay more attention to diet, nutrition, and health. Because luffa is nutritious, delicious, and has beneficial effects on human health, the land area on which it is cultivated continues to increase [1–3]. There are currently two luffa species, namely towel gourd (*Luffa cylindrica* Roem.) and sinkwa towelsponge gourd (*Luffa acutangula* Roxb.). The former is affected by flesh browning, which considerably influences its flavor, odor, nutritional value, and shelf life [4,5]. Consequently, luffa browning has gradually become a prominent topic of interest among researchers. The mechanism regulating the browning of fruits and vegetables has been investigated for several decades, with differences detected in the enzymatic browning of diverse crops. Researchers have been unable to inhibit the enzymatic browning mechanism, and the luffa browning mechanism remains uncharacterized [6,7].

The browning of fruits and vegetables results from non-enzymatic and enzymatic processes [8,9]. Non-enzymatic browning, which involves many types of chemical processes, such as the Maillard reaction, caramelization, vitamin C oxidation, and polyphenol polymerization, is a major cause of browning [10,11]. Enzymatic browning in fresh-cut products is generally considered a multi-factorial process [12,13]. Polyphenol oxidase (EC 1.10.3.1, PPO) encoded by *PPO* multigene family members is a major inducer of the enzymatic browning of fruits, vegetables, and fresh horticultural products, following bruising, cutting, or other cellular damages [14,15]. Many other enzymes, including peroxidase (E1.11.1.7, POD), phenylalanine ammonia lyase (EC 4.3.1.24, PAL), superoxide dismutase (EC1.15.1.1, SOD), and catalase (EC 1.11.1.6, CAT), are also involved in the complex processes that induce or inhibit enzymatic browning of fresh-cut foods [16–18]. The expression levels of the genes encoding these enzymes likely change during the enzymatic browning of fresh-cut fruits and vegetables. Furthermore, the transcriptome profiles from recent studies revealed that the expression levels of genes associated with ethylene, abscisic acid, and gibberellin metabolism were affected by the browning of pears and apples [19,20]. However, little information is available concerning the mechanisms underlying the browning of fresh-cut luffa fruits and how to prevent it.

High-throughput transcriptome sequencing technology has recently been widely used to analyze the gene expression levels of whole organisms. The application of this technology enables the comprehensive study of gene expression during specific states or conditions [21,22]. Additionally, it can be used to identify important functional genes and characterize the molecular mechanisms regulating biological traits. In this study, we used high-throughput sequencing technology to examine gene expression profiles in fresh-cut luffa fruits. We annotated and classified the functions of Unigenes, and analyzed the browning-associated metabolic pathways. Our objective was to clarify the molecular basis of the browning of fresh-cut luffa fruits.

## Materials and methods

### Plant materials

Luffa ‘Fusi-3’ plants were grown in a greenhouse at the Vegetable Research Institute of the Fujian Academy of Agricultural Sciences. Undamaged fruits of a uniform size were collected at 18 days after pollination. Twenty representative luffa ‘Fusi-3’ fruits were harvested from each tree. The fruit samples were cut into 1-cm-thick slices with a stainless steel knife, and incubated at 25 ± 1°C for 1, 3, and 6 h to induce browning. Samples immediately after slicing were used as controls. The control and treated samples were collected at the same time points. The sliced samples were combined and immediately frozen in liquid nitrogen and peeled before being stored at −80°C.

### RNA preparation, library construction, and RNA sequencing

Total RNA was extracted, mRNA was purified, and cDNA libraries were constructed by Guangzhou Gene Denovo Biological Technology Co., Ltd. (Guangzhou, China). The cDNA libraries were constructed as previously described [23] and then sequenced using the HiSeq™ 2500 system (Illumina Inc., San Diego, CA, USA).

The total RNA used for a quantitative real-time polymerase chain reaction (qRT-PCR) assay was extracted from each sample using the EZNA Plant RNA Kit (Bio-tek, Beijing, China). The quantity and quality of the RNA samples were assessed using the NanoDrop ND-1000 spectrophotometer (Thermo Scientific, CA, USA) and a 2100 Bioanalyzer (Agilent Technologies, Santa Clara, CA, USA). The libraries were then sequenced using the HiSeq™ 2000 system (Illumina Inc.). Equal amounts of RNA from three samples collected at the same browning time point were combined.

### Determination of total phenol content

The total phenol content was determined with the Folin–Ciocalteu assay, using a modified version of the method described by Dewanto et al. (2002) [24]. The absorbance (750 nm) of a 25-μL aliquot of extract was recorded, and the total phenol concentration was expressed as gallic acid equivalents (mg GAE g^−1^ fresh weight) based on a calibration curve (1–6 μg mL^−1^ gallic acid). Each biological replicate was measured three times.

### *De novo* transcriptome assembly and functional annotation

The RNA sequencing (RNA-seq) raw data were filtered by removing adapter sequences with in-house Perl scripts to obtain high-quality reads. Reads with low-quality regions (reads with a base quality < 20) were excluded using the sliding window trimming approach. The remaining reads were *de novo* assembled using Trinity software (http://trinityrnaseq.github.io/) [25], with min_kmer_cov set to 2 by default and all other parameters also set to their default values.

We used the luffa Unigene sequences to search the National Center for Biotechnology Information nonredundant (NCBInr) protein, Gene Ontology (GO), Eukaryotic Orthologous Groups of proteins (KOG), Kyoto Encyclopedia of Genes and Genomes (KEGG), and Swiss-Prot databases. Protein functional annotations were based on a BLASTX search (*E* < 10^−5^), which was used to identify the most similar proteins. The GO terms were assigned to Unigenes using Blast2GO [26]. The distribution of the GO functional classifications for the Unigenes was plotted using WEGO software [27]. We identified the genes potentially important for luffa browning using the aforementioned databases.

### Analysis of Unigene expression levels

Reads from four cDNA libraries were mapped to the assembled Unigenes using Bowtie [28]. A fragments per kilobase of exon model per million mapped reads (FPKM) fold-change > 2 and a false discovery rate (FDR) < 0.05 were used as the thresholds to identify significant differences in gene expression [29]. Expression-level differences between two samples were examined using the DEGseq R package [30]. A fold-change ≥ 2 (in both directions; log_2_ ratio ≥ 1) and FDR ≤ 0.05 were used as standards to determine the significance of gene expression level differences among samples. A time series cluster analysis, which is based on the short time-series expression miner (STEM) method (http://www.cs.cmu.edu/~jernst/stem/) [31], was used to identify global trends and similar temporal model expression patterns among all differentially expressed genes (DEGs).

### Quantitative real-time polymerase chain reaction analysis

The RNA-seq data were validated by qRT-PCR using gene-specific primers. Total RNA was extracted from fruits, and a 2-μg sample was used to prepare cDNA with the PrimeScript™ II 1st Strand cDNA Synthesis kit (Takara). The resulting cDNA was used as the template for qRT-PCR, which was conducted using a real-time PCR plate (Applied Biosystems, Foster City, CA, USA), SYBR green as the fluorescent reagent, and an ABI 7500 Fast Real-time PCR system (Applied Biosystems). A standard curve for each target gene was constructed based on qRT-PCR data from a series of diluted cDNA samples. The 20-μL qRT-PCR solutions consisted of 2 μl cDNA, 10 μL SYBR Premix Ex Taq™ II, 6.8 μL EASY Dilution buffer, and 0.6 μL 10 μM forward and reverse primers. The PCR was conducted with the following program: 95°C for 30 s; 40 cycles of 95°C for 10 s and 60°C for 34 s. The *18S rRNA* gene (GenBank accession: KM656452), which was stably expressed according to the RNA-seq data, was used to normalize expression levels [32]. The 2^−ΔΔCt^ method was used to calculate relative gene expression levels with Microsoft Excel software. The qRT-PCR experiment consisted of three independent biological replicates, and the expression levels calculated for each sample were based on three technical replicates.

## Results

### Determination of total phenol content

**Fig 1:**
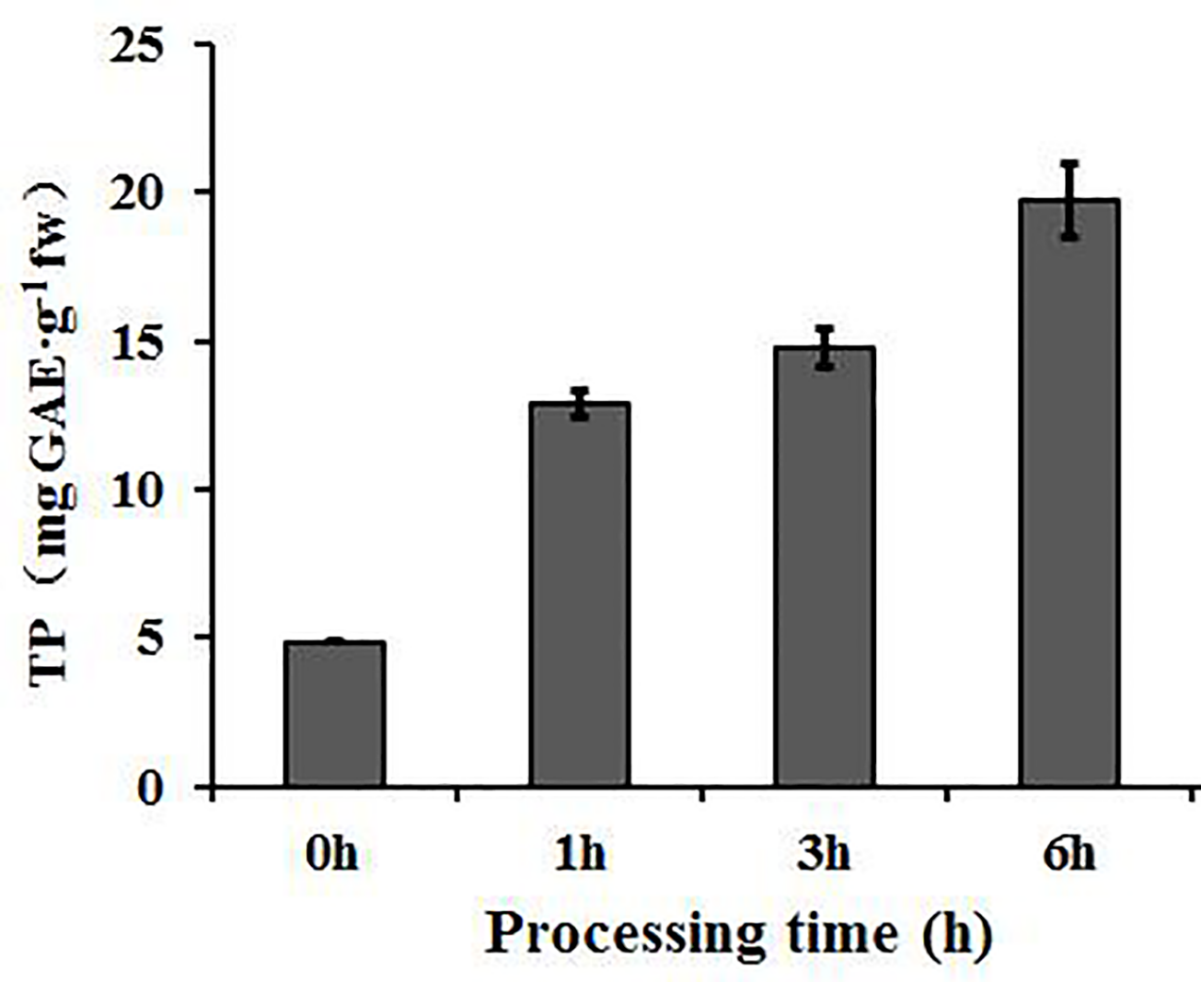
Total phenol content in luffa fruit slices at different browning time points. The vertical bars represent the standard error of triplicate experiments.

Previous studies indicated that among *Luffa cylindrica* cultivars, ‘Fusi-3’ produces fruits that are the most susceptible to browning [33]. Additionally, the total phenol content of browning fresh-cut vegetables under constant temperatures tends to increase over time [13,34]. For a comprehensive transcriptome analysis of luffa fruits, total RNA was extracted from peeled ‘Fusi-3’ fruit slices. The total phenol content exhibited an increasing trend during the browning treatment period (i.e., 0–6 h) (**Fig 1**).

### RNA sequencing and assembly of reads

To analyze the transcription profiles of luffa fruits undergoing browning, four cDNA libraries were created from ‘Fusi-3’ fruits at 18 days after pollination, and analyzed using the Illumina HiSeq™ 2000 system. We generated about 92 million sequence reads. After removing adapter sequences and low-quality reads, we obtained 91 million valid sequencing reads, with an average length of 90.2 bp. After a *de novo* assembly analysis, 58,073 Unigenes were obtained, with an average length of 896.93 bp and an N50 of 1,510 bp. Additionally, 29.86% of the Unigenes were > 1,000 bp long (**Table 1**).

**Table 1:** Summary of the transcriptome during the browning of fresh-cut luffa fruits.

**Sequences**	**0h**	**1h**	**3h**	**6h**
**BEFORE TRIMMING**				
**Total nucleotides (bp)**	2,998,625,250	2,760,038,000	3,010,284,000	2,732,343,750
**Number of raw reads**	23,989,002	22,080,304	24,082,272	21,858,750
**AFTER TREMMING**				
**Number of clean reads**	23,505,162	21,668,306	23,622,892	21,429,916
**GC content (%)**	45.81	46.29	46.08	47.16
**Q30 percentage (%)**	94.74	94.77	94.83	94.73
**AFTER ASSEMBLY**				
**Number of Unigenes of combined data**	58,073			
**Total nucleotides (nt) of Unigenes (bp)**	52,087,451			
**Mean length of Unigenes (bp)**	896.93			
**Sequence length > 1000 bp (%)**	29.86			
**N50 of Unigenes (bp)**	1510			

### Sequence annotation

**Fig 2:**
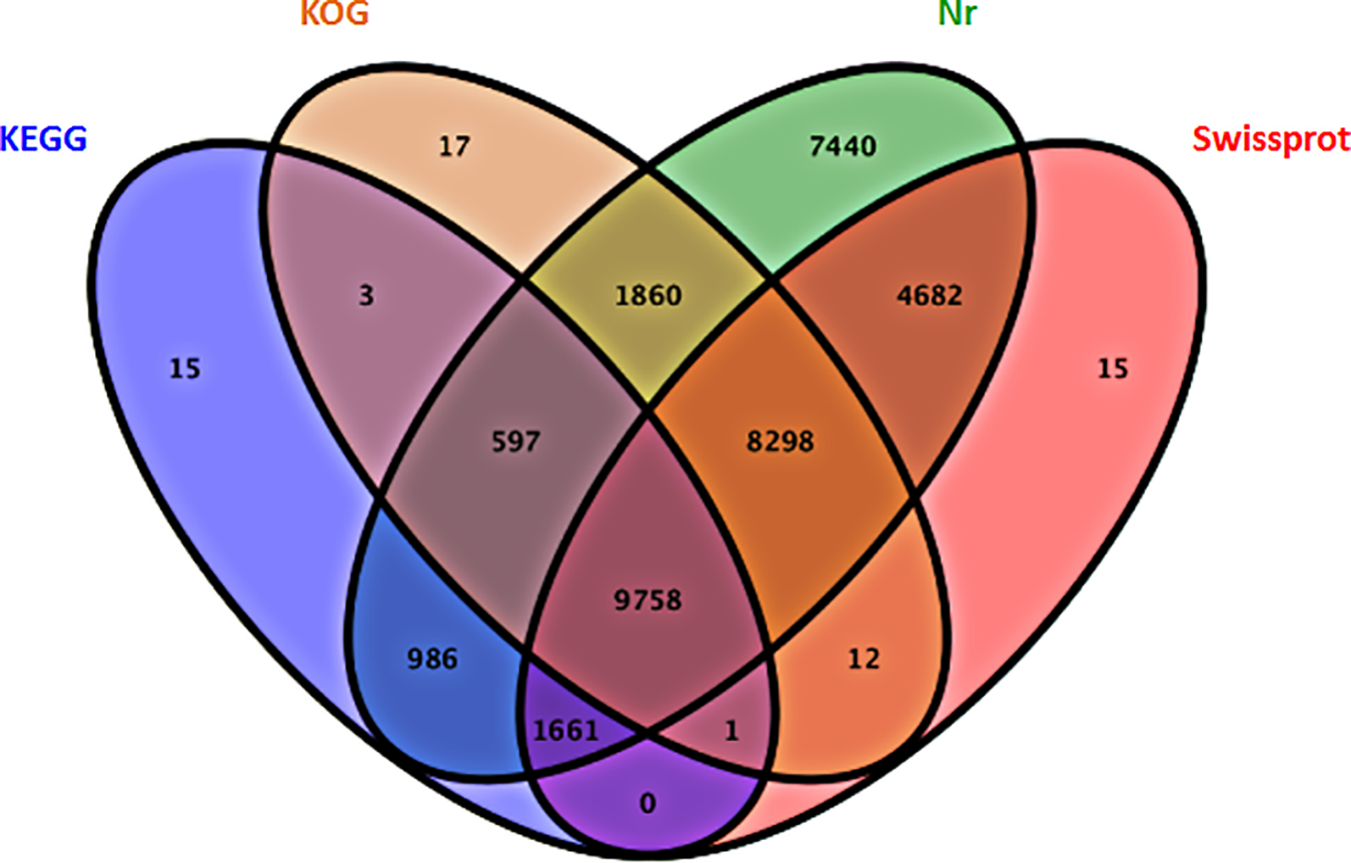
Unigenes annotated by a BLASTX search of databases (*E* < 10^−5^).

The annotation of Unigenes provided information regarding functions, protein sequence similarities, and KOG, GO, and KEGG pathway details. The data were screened against the following four public databases: NCBInr, Swiss-Prot, KOG, and KEGG. Unigenes were annotated according to the best BLASTX matches (*E* < 10^−5^) and sequence identities > 30%. A total of 35,345 (60.86%) Unigenes were annotated. The assembled sequences included 37,321 (60.75%), 24,427 (30.87%), 20,546 (30.52%), and 13,021 (18.83%) Unigenes with matches in the NCBInr, Swiss-Prot, KOG, and KEGG databases, respectively (**S1 Table and Fig 2**).

**Fig 3:**
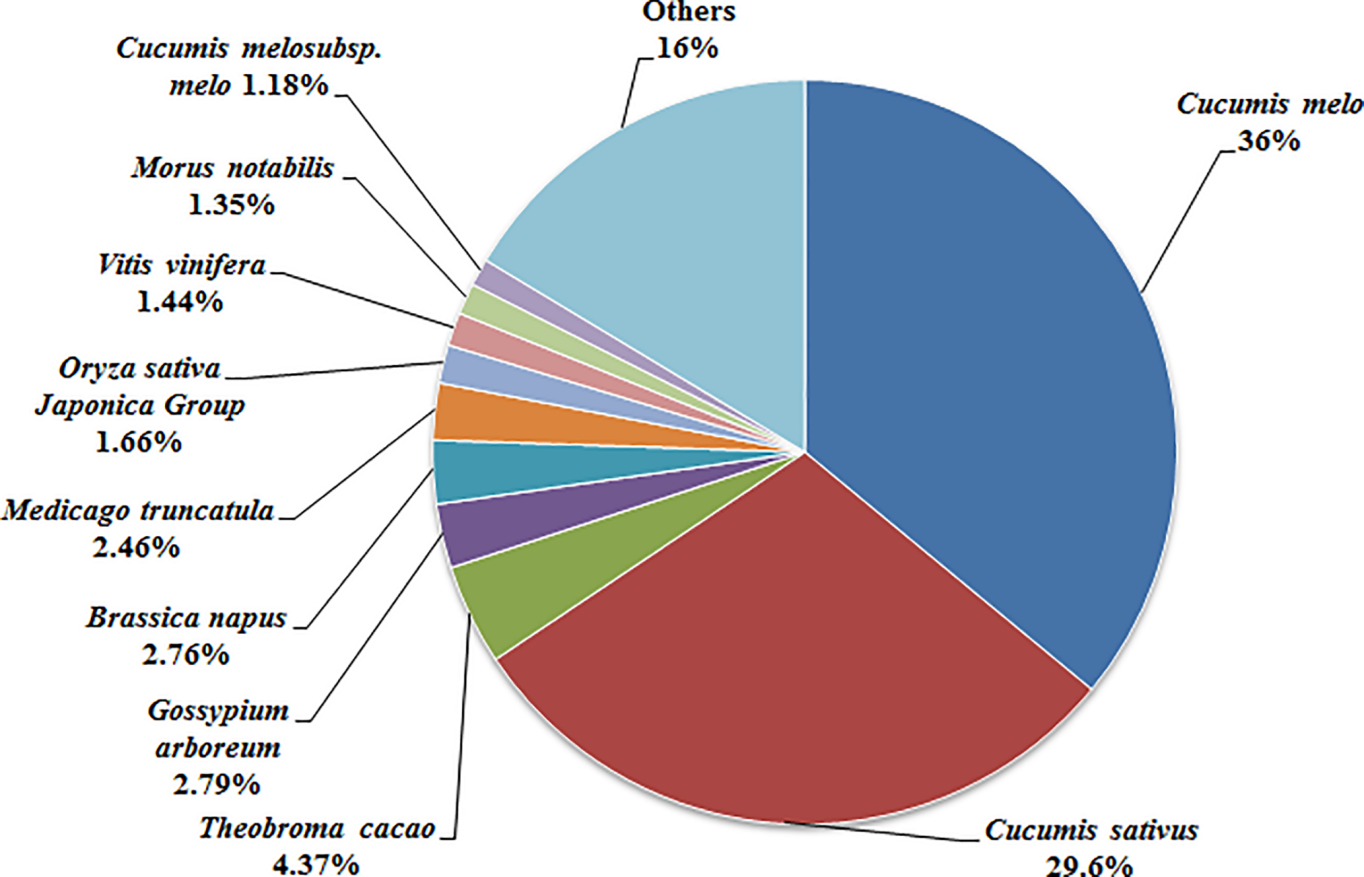
Unigenes with matches in the NCBInr database and homologs in 10 plant species.

The most abundant BLAST hits were associated with plant species, and 35,282 (60.75%) of the Unigenes with matches in the NCBInr database had homologs in 10 plant species. The five species with the most hits were *Cucumis melo* [12,702 (36%)], *Cucumis sativus* [10,445 (29.6%)], *Theobroma cacao* [1,543 (4.37%)], *Gossypium arboreum* [983 (2.79%)], and *Brassica napus* [973 (2.76%)] (**Fig 3**). The fact that the luffa Unigenes had the most matches with *C*. *melo* sequences suggested that *C*. *melo* may be a useful reference material for further analyses of luffa functional genes. A total of 27,858 Unigenes were annotated by one or more databases (i.e., 78.81% of all assembled Unigenes), implying they have relatively well conserved functions.

**Fig 4:**
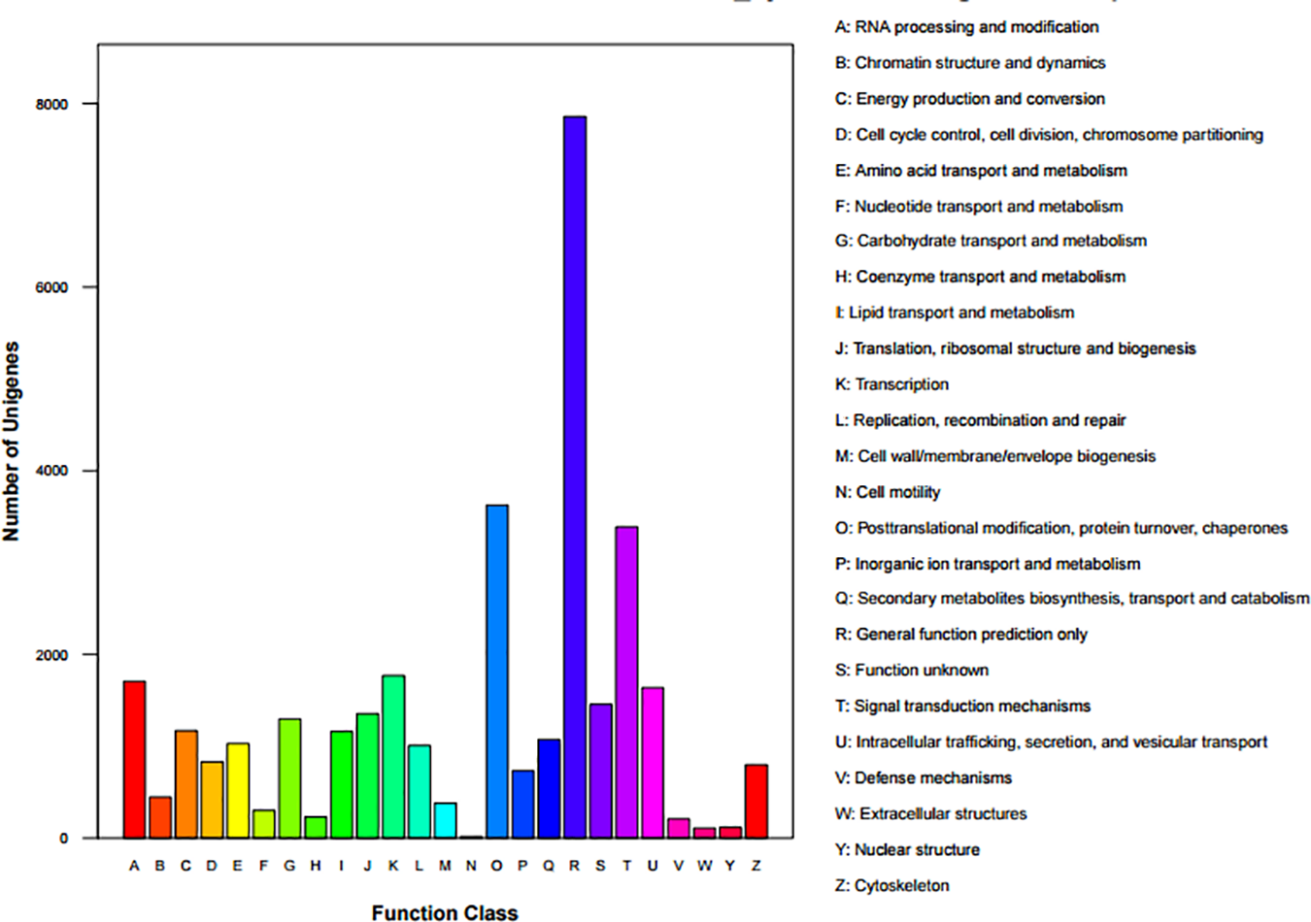
Histogram of the KOG classifications.

The luffa Unigenes were searched against the KOG database to predict and classify their possible functions. Of 37,321 NCBInr matches, 31,567 sequences had KOG classifications among 25 KOG categories (**Fig 4**). The most common categories were “general function prediction only”, with 7,857 Unigenes, and “posttranslational modification, protein turnover, and chaperones” (3,626 Unigenes). “Cell motility” had the fewest Unigenes, with only 17. Our data revealed that annotated Unigenes were associated with most of the activities related to growth and development.

**Fig 5:**
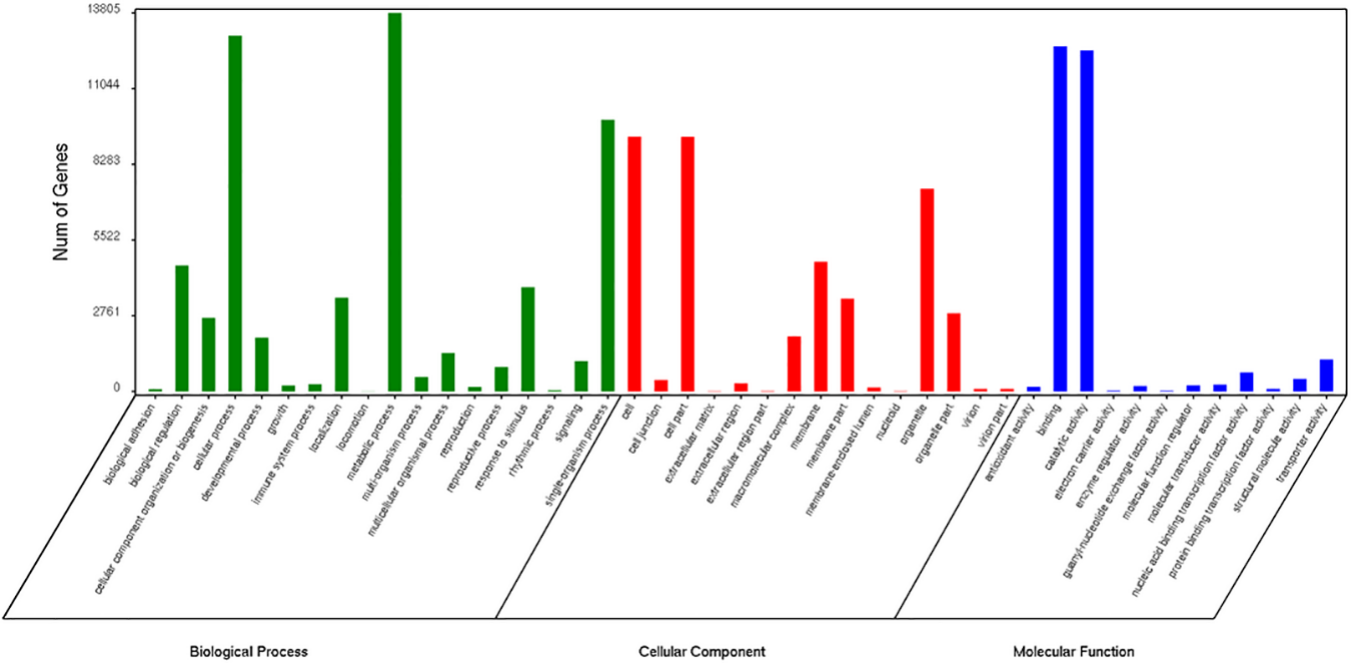
Summary of the GO classification of Unigenes.

The GO assignments were used to classify the functions of the luffa transcripts. Based on sequence homologies, the 24,021 annotated sequences with BLAST hits to PlantGDB proteins were categorized into 45 functional groups (**Fig 5**). “Metabolic process”, “cellular process”, and “binding” were the dominant terms in the “biological process”, “cellular component”, and “molecular function” main categories, respectively. We also identified a relatively large number of genes associated with “catalytic activity”, “single-organism process”, and “cell”, with only a few genes related to “extracellular region part”, “nucleoid”, “extracellular matrix”, and “locomotion”.

**Fig 6:**
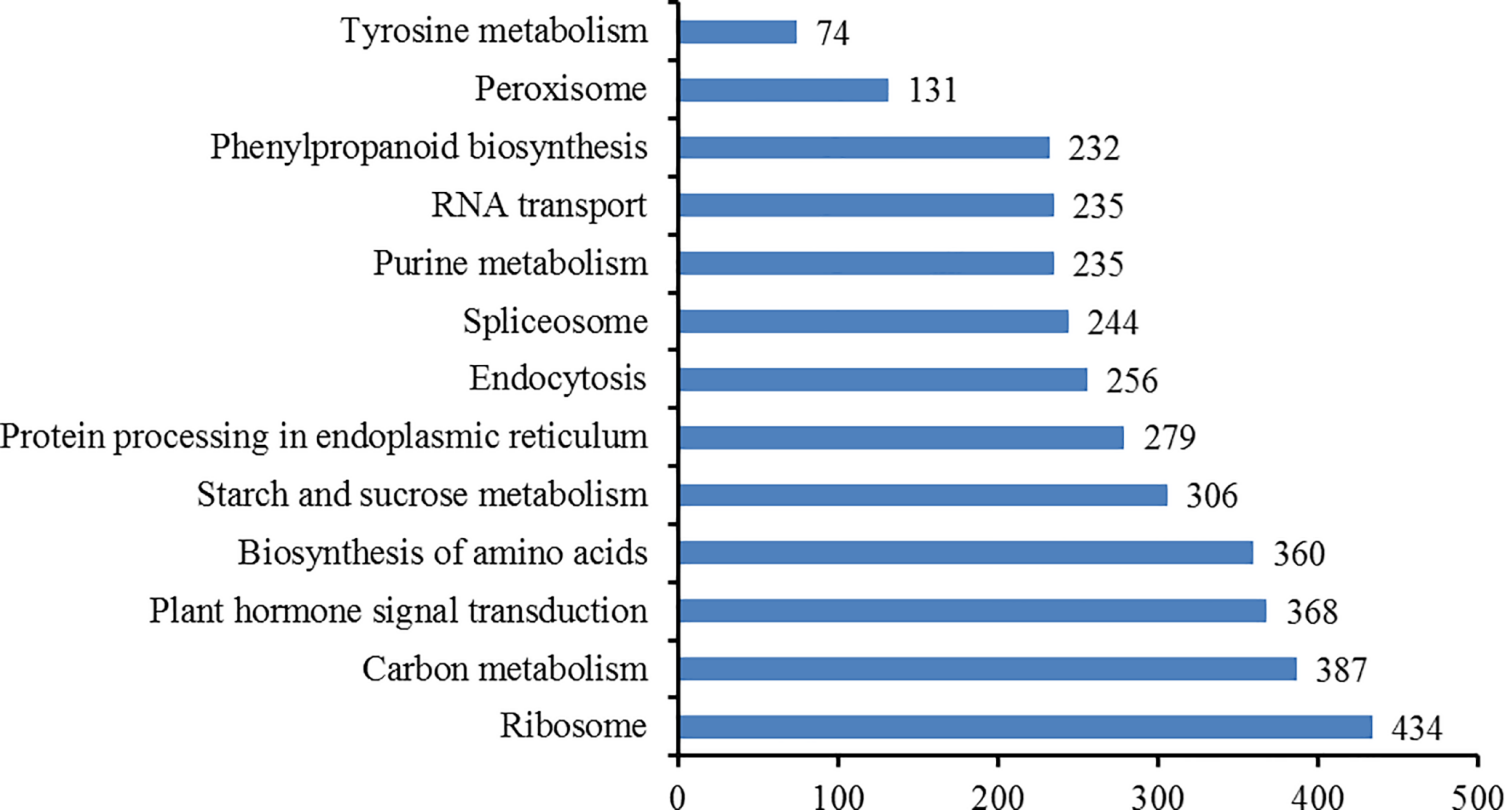
Pathway assignments based on the KEGG database.

Pathway-based analyses may be useful for clarifying the biological functions of genes. The KEGG pathway database contains information regarding networks of intracellular molecular interactions, as well as their organism-specific variations [35]. To identify the biological pathways that are active during the browning of fresh-cut luffa fruits, we mapped the 58,073 Unigenes to the reference canonical pathways in the KEGG database. A total of 13,021 Unigenes were annotated (**S2 Table**). The top 10 pathways (i.e., “ribosome”, “carbon metabolism”, “plant hormone signal transduction” “biosynthesis of amino acids”, “starch and sucrose metabolism”, “protein processing in endoplasmic reticulum”, “endocytosis”, “spliceosome”, “purine metabolism”, and “RNA transport”) and three potential browning-related pathways (i.e., “phenylpropanoid biosynthesis”, “peroxisome”, “tyrosine metabolism”) are presented in **Fig 6**. Moreover, there were 47, 131, and 232 Unigenes associated with the “tyrosine metabolism”, “peroxisome” and “phenylpropanoid biosynthesis” pathways, respectively. These results indicated that diverse metabolic processes are active in luffa fruit flesh, resulting in the synthesis of diverse metabolites. These annotations may serve as a valuable resource for investigating specific processes, functions, and pathways, and facilitate the identification of novel genes involved in the browning of luffa fruit flesh.

### Analysis of differentially expressed genes

**Fig 7:**
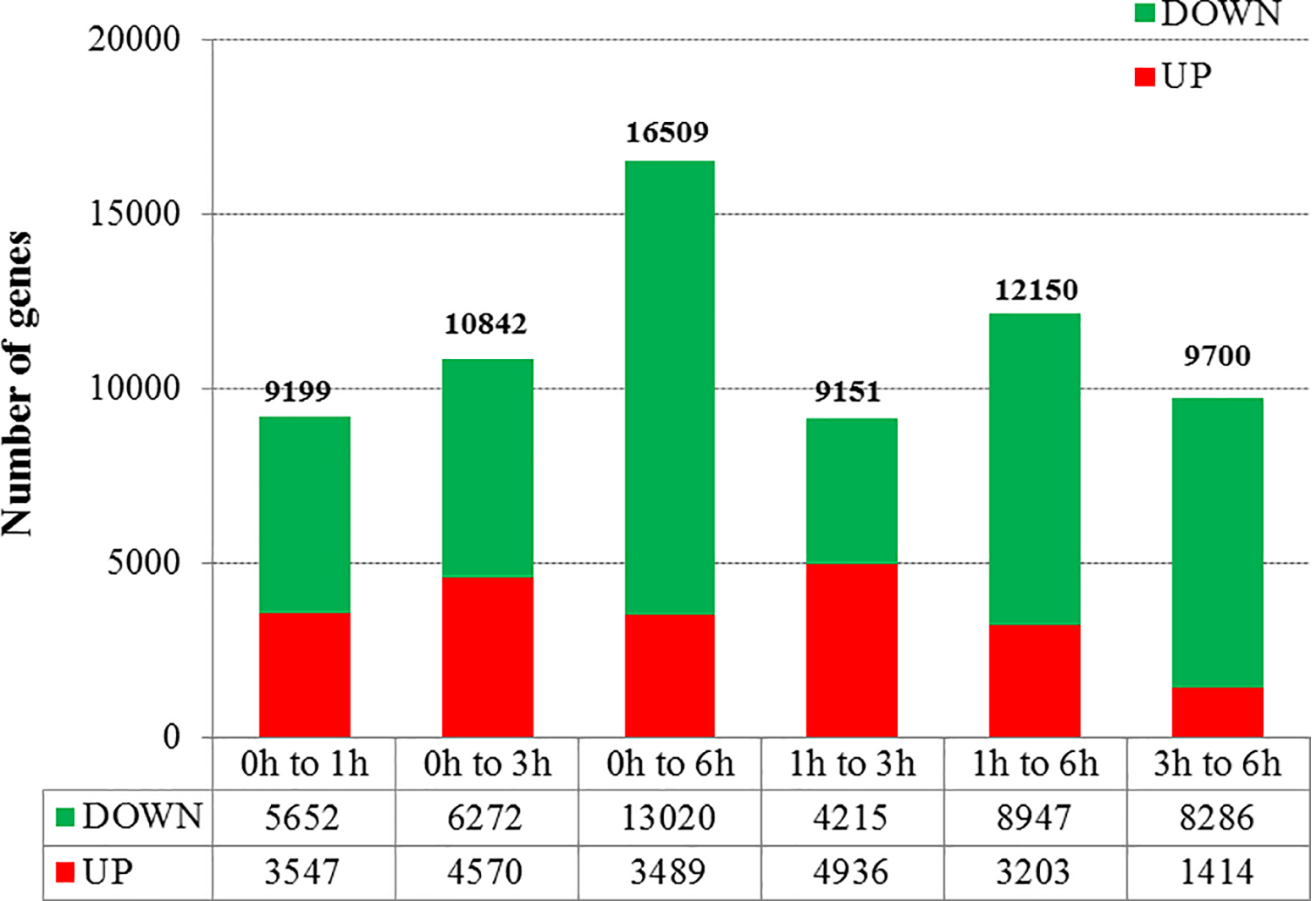
Differentially expressed genes in fresh-cut luffa fruits at various time points.

**Fig 8:**
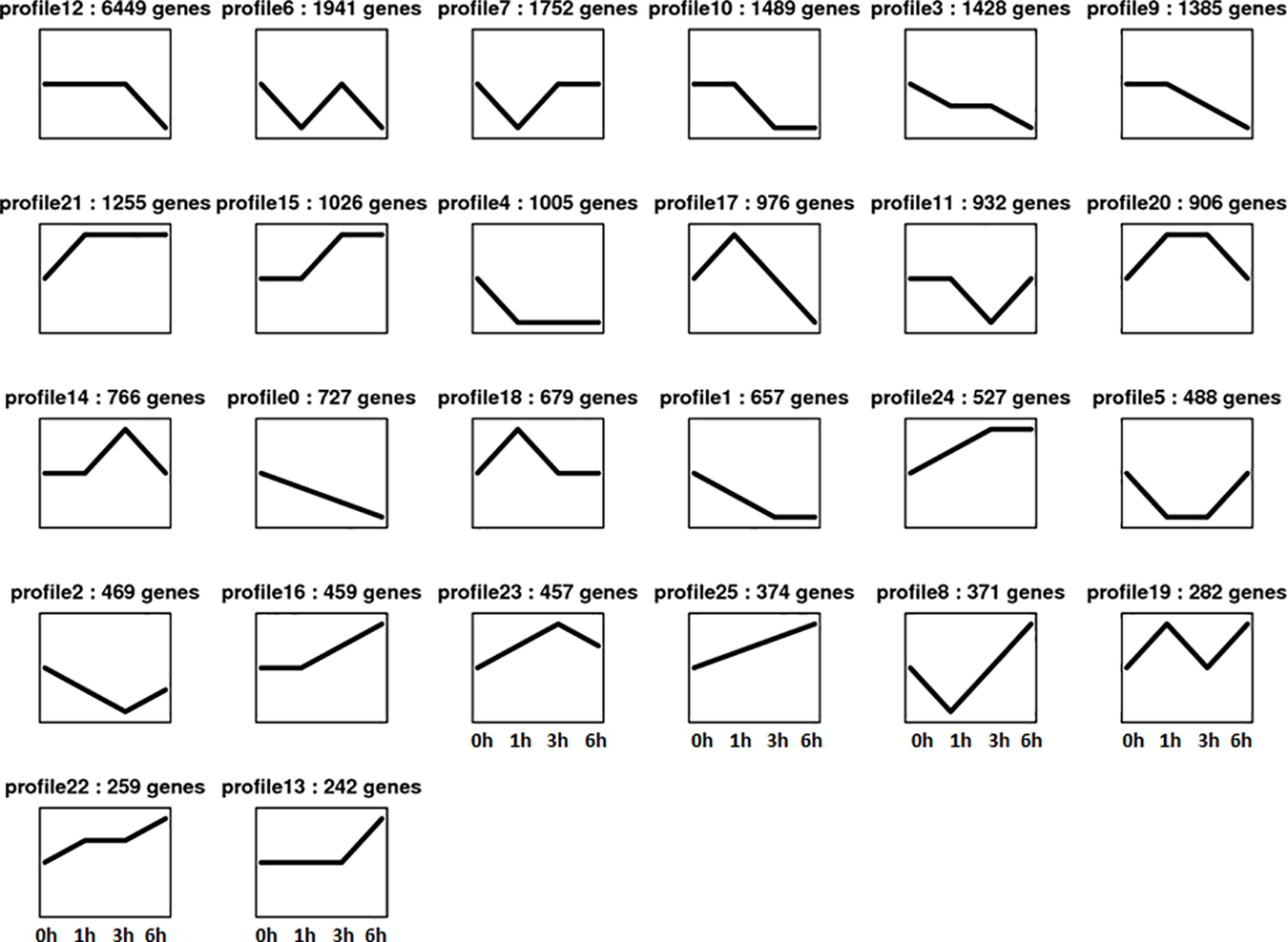
Clustering and classification of 27,301 differentially expressed genes. The numbers in the top corner of each panel represent the identification number of the 26 profiles and the number of identified genes in the cluster, respectively.

Based on a published protocol, we developed an algorithm to identify genes that were differentially expressed between two samples [36]. The FDR was used to determine the P-value threshold in multiple analyses for calculating the expression-level differences between two samples [37]. We calculated the number of expressed sequence tags corresponding to each gene in each library to estimate gene expression levels. A total of 27,301 Unigenes were differentially expressed during the browning of luffa fruits (**S3 Table**). The highest number of DEGs was detected during the comparison between the 0 and 6 h time points [i.e., 16,509 (3,489 up- and 13,020 down-regulated at 6 h) (log_2_ ratio ≥ 1; FDR ≤ 0.05; P ≤ 0.01)] (**Fig 7**). We used the STEM method to refine the sets of genes that were differently expressed at a minimum of four time points (i.e., 0, 1, 3, and 6 h). This method is commonly used for gene clustering in transcriptomic studies [31]. A total of 27,301 DEGs were clustered into 26 possible model profiles based on expression patterns (**Fig 8**). The DEGs were then subjected to GO and KEGG pathway enrichment analyses.

### Identification of potential browning-related genes

We selected DEGs with an FDR ≤ 0.05 and an expression ratio > 2 for GO functional and KEGG pathway analyses (**S4 Table**). We used the MapMan tool to visualize the annotated processes of interest or metabolic pathways. We conducted pathway enrichment analyses to identify significantly enriched metabolic or signal transduction pathways associated with the DEGs [38]. According to GO functional annotations and the screening of 58,073 Unigenes, we identified 11 Unigenes with potential roles in the browning of fresh-cut luffa fruits, including three *PPO* genes (Unigene0043875, Unigene0050171, and Unigene0015096), two *PAL* genes (Unigene0044748 and Unigene0024978), one *POD* gene (Unigene0011770), two *CAT* genes (Unigene 0036262 and Unigene0033876), and three *SOD* genes (Unigene0021782, Unigene0008835, and Unigene0015506), as well as four WRKY transcription factors (S1 Table). The Unigenes were annotated based on the NCBInr protein database and divided into the following categories: oxidoreductase, ammonia lyase, antioxidant, catalytic activity, and DNA-binding function related. Furthermore, a KEGG analysis revealed that 15 genes may affect tyrosine metabolism, phenylpropanoid biosynthesis, or the peroxisome pathway (**S4 Table**). Additionally, with the aid of rapid amplification of cDNA ends technology, we obtained the full-length *CAT* (Unigene0036262) sequence, which has been deposited in the GenBank database (**Table 2**).

**Table 2:** Unigenes potentially associated with the browning of luffa fruits.

**Unigene ID**	**Nuleotide** **length (bp)**	**Full** **length**	**Protein** **Length (aa)**	**Homologous function** **in nr**	**Homology species &** **Accession number**	**E-value**	**GenBank** **accession numbers**
**Unigene0043875**	2,189	Yes	589	Polyphenol oxidase, chloroplastic-like	*Cucumis melo* XP_008442274.1	0.0	KM506756
**Unigene0050171**	1,990	Yes	574	Polyphenol oxidase, chloroplastic-like	*Cucumis melo* XP_008442274.1	0.0	KR819890
**Unigene0015096**	2,189	Yes	593	PREDICTED: polyphenol oxidase, chloroplastic-like	*Cucumis melo* XP_011047843.1	0.0	KX092429.1
**Unigene0044748**	2,365	Yes	715	Phenylalanine ammonia-lyase 1	*Cucumis sativus* XP_004143255.1	0.0	KP341758
**Unigene0024978**	2,274	Yes	713	Phenylalanine ammonia-lyase-like	*Cucumis sativus* XP_004145752.1	0.0	KR491944
**Unigene0011770**	1,319	Yes	331	Peroxidase 2-like	*Cucumis melo* XP_008449774.1	0.0	KM506755
**Unigene0036262**	1,259	5′	492	Catalase isozyme 1	*Cucumis melo* XP_008452956.1	0.0	KP222260
**Unigene0033876**	1,709	Yes	492	Catalase	*Cucurbita moschata* AHF27430.1	0.0	KR184674
**Unigene0021782**	1,775	Yes	152	Superoxide dismutase [Cu-Zn]-like	*Cucumis sativus* NP_001267697.1	5E-89	KP178922
**Unigene0008835**	799	Yes	157	PREDICTED: superoxide dismutase [Cu-Zn] 2	*Cucumis sativus* XP_004147539.1	2E-98	KX092445.1
**Unigene0015506**	1,011	Yes	221	PREDICTED: superoxide dismutase [Cu-Zn]	*Cucumis sativus* XP_008465422.1	7E-131	KX092446.1
**Unigene0018509**	2,189	Yes	299	probable WRKY transcription factor 69-like	*Cucumis sativus* NM_001280699.1	5E-172	KY621843
**Unigene0021412**	1,990	Yes	616	PREDICTED: probable WRKY transcription factor 31	*Cucumis sativus* XM_004134727.2	0.0	KY621844
**Unigene0025291**	2,365	Yes	435	PREDICTED: probable WRKY transcription factor 31	*Cucumis sativus* XM_011660396.1	0.0	KY621845
**Unigene0034271**	2,274	Yes	388	PREDICTED: probable WRKY transcription factor 7 isoform X2	*Cucumis melo* XM_008454358.2	0.0	KY621846
**Unigene0019122**	1,273	Yes	178	PREDICTED: ethylene-responsive transcription factor ERF109-like	*Cucumis melo* XP_008454945.1	2E-93	MF678591
**Unigene0003760**	1,012	Yes	119	PREDICTED: abscisic acid receptor PYL8-like	*Cucumis melo* XP_008461524.1	6E-120	MF678592
**Unigene0031055**	1,111	Yes	205	Gibberellin 2-oxidase	*Cucurbita maxima* XP_008439633.1	0.0	MF678593

### Validation of differentially expressed genes based on RNA-seq data by quantitative real-time PCR

**Fig 9:**
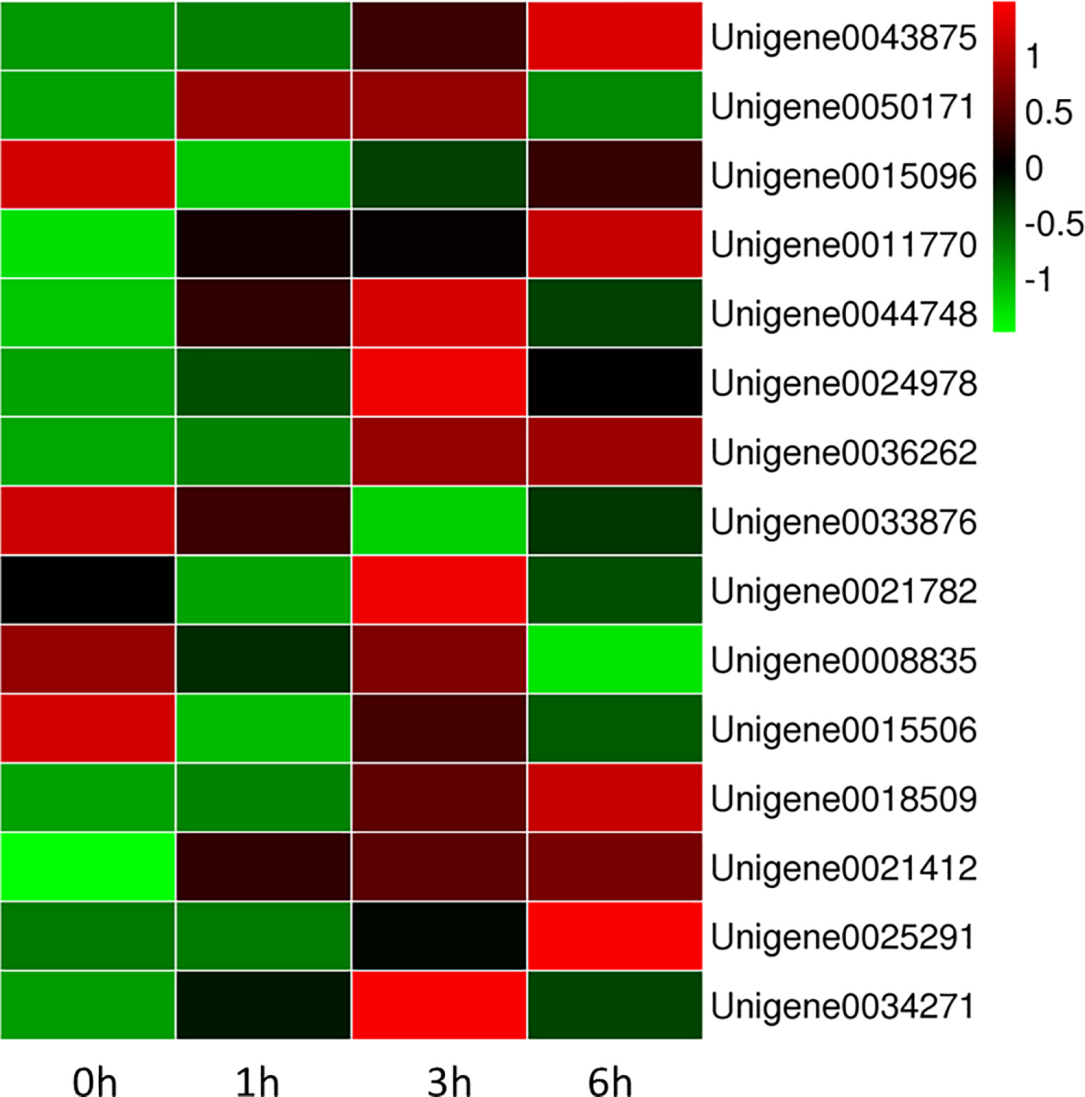
Clustering of 15 browning-related genes. The color scale represents FPKM-normalized log_2_-transformed gene expression levels. Different columns represent different samples, and different lines represent different genes.

**Fig 10:**
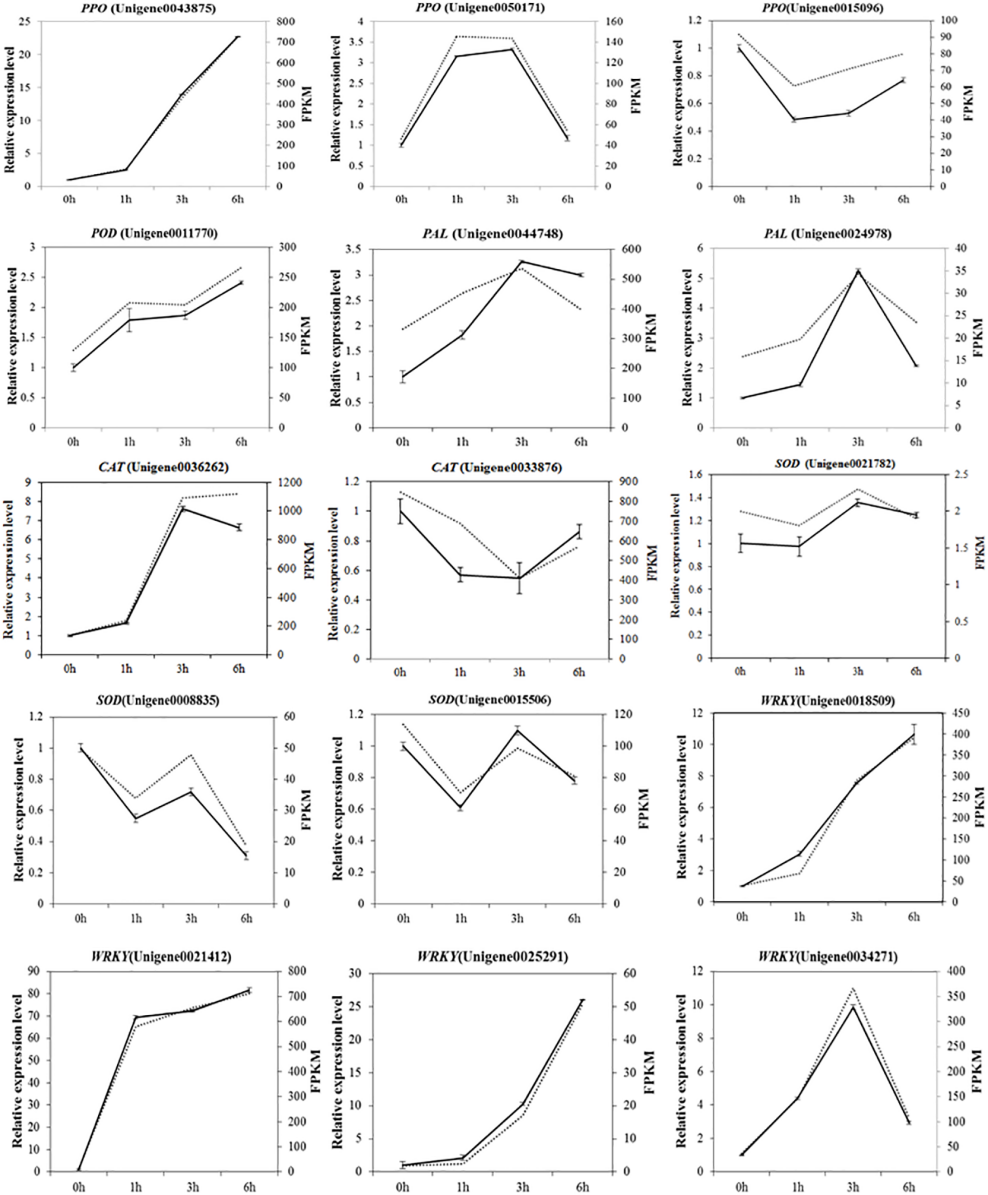
Quantitative real-time polymerase chain reaction analyses of the expression levels of selected Unigenes during the browning of luffa fruits. *18S rRNA* was used as the internal control. The error bars represent the standard error of three biological replicates.

**Fig 11:**
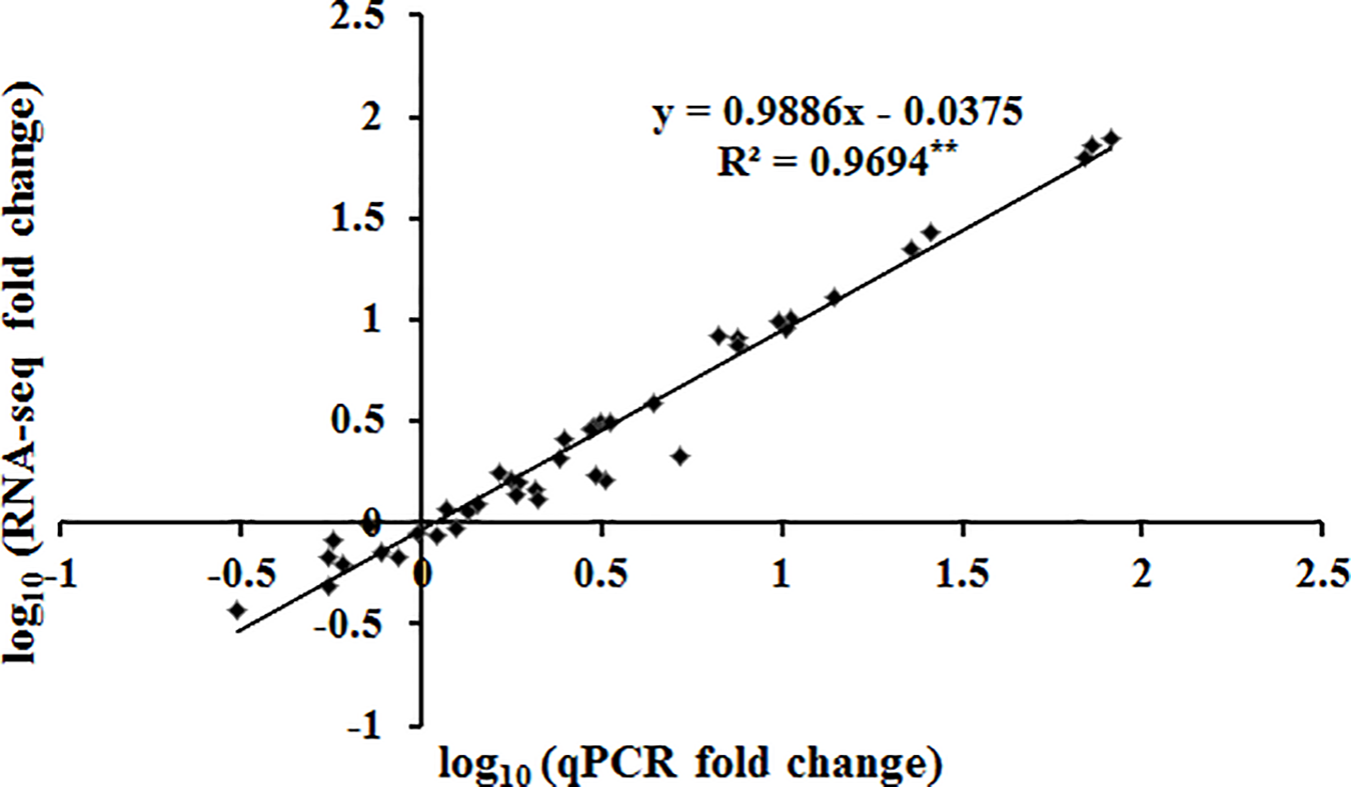
Correlation analysis of fold-change values based on RNA-seq and qRT-PCR data. The RNA-seq fold-change refers to the ratio of FPKM values from 0 to 6 h. The qRT-PCR fold-change refers to the expression levels at 1, 3, and 6 h normalized against the expression level at 0 h. **significant correlation (P < 0.01).

The heat maps for the 11 browning-related genes and four WRKY transcription factors indicated that the expression levels varied at different time points (i.e., 0, 1, 3, and 6 h) (**Fig 9**). We used qRT-PCR with gene-specific primers (**Table 3**) to analyze the expression profiles of genes likely involved in the browning of fresh-cut luffa fruits. The expression levels were mainly up-regulated relative to the 0 h expression levels, except for *SOD* (Unigene0021782), which was down-regulated at the 1 h time point, and *PPO* (Unigene0015096), *CAT* (Unigene0033876), and *SOD* (Unigene0008835 and Unigene0015506), which were down-regulated at the 1, 3, and 6 h time points (**Fig 10**). A linear regression analysis revealed a strong positive correlation (R^2^ = 0.9694; P ≤ 0.01) between the fold-change values based on qRT-PCR and RNA-seq data (**Fig 11**). These results confirm the reliability of the RNA-seq data generated in this study.

**Table 3:** Details regarding the qRT-PCR primers used to analyze 15 genes.

Unigene ID	Directions	Primers	Product size	TM (°C)	Relative Expression Level
Unigene0043875	Forward	CGAATGTTCACTGTGCCTAT	191	60	up
	Reverse	CTTCGGAGCGTCGTAGT			
Unigene0050171	Forward	CTAGCCGTGGAAACCGA	218	62	up
	Reverse	TGATTGGCTCACAGTGGA			
Unigene0015096	Forward	AGTTCCCGATCACTCTTAAT	216	58	down
	Reverse	TCACGAAGCTGCCG			
Unigene0044748	Forward	CGCTGGACCCAACGTACAGG	195	66	up
	Reverse	GGTTGACAGTGTCGACTGTGTCC			
Unigene0024978	Forward	GCTCAATTTTCTGAGCTTGT	169	59	up
	Reverse	GGACGTGGCTTGTTAC			
Unigene0011770	Forward	GCCAATGTACTTGCTCTTCT	199	60	up
	Reverse	GGTCCAACTCATGTAACTTCT			
Unigene0036262	Forward	CTAGTGGAGAAACTTGCTAACT	189	59	up
	Reverse	GGATAACAGTGGAGAAACGT			
Unigene0033876	Forward	TCACCATAACAACCACCATGAAG	152	62	down
	Reverse	CACACACCTTTCTCTCTTTCCG			
Unigene0021782	Forward	CACAGGAAAGATGGTGAAGG	210	60	up
	Reverse	CCAGCAGGGTTGAAATGT			
Unigene0018509	Forward	CATATCCACCGTCCGATTC	214	59	up
	Reverse	TGGTGGTGAGATTTGGTG			
Unigene0021412	Forward	CTAATGAACCCAAATTTATTGGCT	165	60	up
	Reverse	GAAGGGTACGTGGAACG			
Unigene0025291	Forward	CAGTTTATGGATCTTGGATTGG	200	59	up
	Reverse	GAAGAAGAAGACGAAGAACTG			
Unigene0034271	Forward	CATCACTCCGAAGACCATTC	172	60	up
	Reverse	GGGAAGTAGTAGTAACAACGG			
Unigene0008835	Forward	CCACGCTCTTGGCGATACA	75	61	down
	Reverse	CCATGGTCCTTCTTCAATGGA			
Unigene0015506	Forward	CACTTCTCCATAGCAAATGC	127	59	down
	Reverse	GGTCAGGGAAGGCG			
18SrRNA	Forward	GTGTTCTTCGGAATGACTGG	271	60	-
	Reverse	ATCGTTTACGGCATGGACTA			

## Discussion

Luffa is an important vegetable crop worldwide. It is rich in nutrients and medicinal compounds, including carotenoids, β-carotene, and α-carotene, which maintain the immune system [39]. To identify genes involved in the browning of luffa fruits, total RNA was extracted from ‘Fusi-3’ fruits and used for mRNA preparation, fragmentation, and cDNA synthesis. The cDNA was sequenced using the Illumina Genome Analyzer IIx platform, and the resulting sequencing data were subjected to bioinformatic analysis. We generated 92,010,328 raw reads from a 200-bp insert (mean size) cDNA library. After trimming adapter sequences and removing low quality reads, 90,226,276 clean reads were obtained. Of these clean reads, 94.19% were considered high-quality reads, which were analyzed further. We annotated 35,345 Unigenes (i.e., 60.86% of the assembled Unigenes), suggesting their functions were relatively conserved. In addition to identifying genes or proteins, we also analyzed the associated GO terms and potential roles in metabolic pathways. Combining our data regarding Unigenes expressed in fresh-cut luffa fruits with the results of a previous transcriptomic study of the same luffa variety reveals that luffa fruits may be a useful source of abundant genes [40]. The current study represents the first attempt at applying Illumina paired-end sequencing technology to investigate the changes in the transcriptome during the browning of fresh-cut luffa fruits.

Enzymatic processes are generally recognized as the main determinants of browning. The accumulation of transcripts for genes associated with the oxidation of phenolic compounds is a hallmark of enzymatic browning. Many studies have confirmed that browning-related genes are differentially expressed in fresh-cut vegetables [41–43]. The *PPO* and *POD* genes have been associated with the browning of fruit and vegetable tissues, and their encoded enzymes function together to induce browning [44,45]. The PPO enzyme encoded by members of the *PPO* multigene family is considered to be a major factor responsible for enzymatic browning of several fresh-cut vegetables [17,46]. Zhou et al. (2015) [47] reported that the enzymatic browning of fresh-cut Chinese chestnut can be inhibited by applying salicylic acid to decrease PPO activity. Their findings provide indirect evidence that PPO influences the browning of fresh-cut Chinese chestnut. Moreover, a previous study revealed that a 3-day treatment with 1 mg L^−1^ ozone decreased POD activity in fresh-cut lettuce, resulting in obviously inhibited browning [48]. Additionally, Chisari et al. (2007) [46] reported that the POD enzyme also affected the browning of fresh-cut melon. In this study, the observed total phenol contents and the results of a KEGG analysis revealed that three *PPO* genes (Unigene0015096, Unigene0043875, and Unigene0015096) and one *POD* gene (Unigene0011770) related to the tyrosine metabolism pathway were differentially expressed while the phenolic acid (i.e., gallic acid) content increased in fresh-cut luffa fruit slices (**S4 Table**) [49,50]. The production of phenolic acids, including gallic acid, L-tyrosine, chlorogenic acid, and gentisic acid, catalyzed by browning-related enzymes (i.e., PPO and POD) can be affected by the tyrosine metabolism pathway [51–54]. However, the exact roles of PPO and POD during responses to the browning of fresh-cut luffa fruits remains to be determined.

In addition to *PPO* and *POD*, the *PAL*, *CAT*, and *SOD* genes are also considered to have important roles in the enzymatic browning of fruits and vegetables [55,56]. The PAL enzyme is reportedly involved in the browning of fresh-cut potatoes [13]. Additionally, a lettuce PAL homolog was observed to be responsible for regulating the browning of fresh-cut edges [57]. Our results indicated that the relative expression levels of two luffa *PAL* genes (Unigene0044748 and Unigene0024978) involved in the phenylpropanoid biosynthesis pathway were up-regulated (**S4 Table**). It is widely accepted that phenylpropanoid biosynthesis pathways are important for controlling the browning of cut vegetables and fruits (**S4 Table**) [58–61]. Additionally, the CAT enzyme has a pivotal role in the browning of fresh-cut fruits and vegetables [62–64]. Dong et al. (2015) [44] proposed that the activity of CAT can be enhanced by a short-term carbon dioxide treatment, leading to inhibited browning of fresh-cut burdock during storage at low temperatures. Meanwhile, Fan et al. (2016) [65] reported that significantly higher activities of antioxidant enzymes (i.e., SOD and CAT) were maintained in browning ‘Laiyang’ pear fruits, although PPO activity was inhibited. Another important enzyme related to the browning of fresh-cut fruits and vegetables is SOD, which is essential for enhancing antioxidant activities in fresh-cut Chinese water chestnut [66]. In this study, two *CAT* genes (Unigene0036262 and Unigene0033876) and three *SOD* genes (Unigene0021782, Unigene0008835, and Unigene0015506) related to the peroxisome pathway were also differentially expressed during the browning of fresh-cut luffa fruits (**S4 Table**).

The browning of fruits and vegetables is caused by the overproduction of reactive oxygen species [67–69]. Members of the WRKY transcription factor superfamily have been detected exclusively in plants, and affect diverse physiological responses and metabolic pathways [70,71]. Previous studies confirmed that WRKY transcription factors are important inducers of the expression of genes associated with the reactive oxygen species pathway during plant responses to biotic, abiotic, and oxidative stresses [72,73]. In this study, we observed that the expression levels of four Unigenes (Unigene0018509, Unigene0021412, Unigene0025291, and Unigene0034271) annotated as WRKY transcription factors were significantly up-regulated at various time points (i.e., 0, 1, 3 and 6 h) during the browning of fresh-cut luffa fruits. Analyses revealed that these four transcription factors (GenBank ID: KY621843–KY621846) were members of the Group II WRKY superfamily, and contained a conserved WRKY domain and C_2_H_2_-type zinc fingers [74]. Furthermore, the current study is the first to reveal a relationship between these four WRKY transcription factors and the browning of luffa fruits. Further studies involving structural and functional characterizations are required to determine whether the identified candidate genes influence the browning of fresh-cut luffa fruits.

Transcriptome profiles of different luffa cultivars showed that the expression levels of *PPO POD* and *PAL* genes related with browning in XTR05 (browning sensitive) were significantly higher than that in YLB05 (browning resistant) [40]. In this study, we also identified three *PPO* genes, one *POD* gene and two *PAL* genes were differentially expressed during the browning of fresh-cut luffa fruits (i.e., after 1–6 h). Moreover, we identified two *CAT* genes, three *SOD* genes and four *WRKY* genes, which were differentially expressed and may play an important role in browning process of fresh-cut luffa fruits. We also observed the differential expression of other Unigenes, such as Unigene0019122, Unigene0003760, and Unigene0031055, which were predicted to encode an ethylene-responsive transcription factor ERF109-like protein, an abscisic acid receptor PYL8-like protein, and gibberellin 2-oxidase, respectively (**Table 2**). These Unigenes may contribute to the browning of fruits and vegetables [40,75,76]. However, their potential effects on the browning of fresh-cut luffa fruits will need to be confirmed in future studies.

## Conclusions

We herein describe the first use of Illumina sequencing technology to investigate the changes in the transcriptome during the browning of fresh-cut luffa fruit slices. A total of 27,301 differentially expressed Unigenes were detected in four sequenced libraries. Additionally, 11 browning-related genes from five gene families (i.e., *PPO*, *PAL*, *POD*, *CAT*, and *SOD*) as well as four WRKY transcription factors were observed to be differentially regulated in fresh-cut luffa fruits. These genetic resources and putative signaling pathways related to luffa defense responses against browning may be useful for future molecular studies of *L*. *cylindrica*.

## Supporting information

S1 Table Unigenes functionally annotated based on the gene ontology database. (XLSX)

S2 Table Unigenes annotated with KEGG pathways. (XLSX)

S3 Table Differentially expressed Unigenes during the browning of luffa fruits. (XLSX)

S4 Table DEGs associated with GO function and KEGG pathways. (XLSX)
